# Curriculum reform: the more things change, the more they stay the same?

**DOI:** 10.1007/s40037-016-0252-x

**Published:** 2016-01-28

**Authors:** Lorraine Hawick, Simon Kitto, Jennifer Cleland

**Affiliations:** Institute of Education for Medical and Dental Sciences, College of Life Sciences and Medicine, University of Aberdeen, Scotland, AB UK; Department of Innovation in Medical Education (DIME), University of Ottowa, Ottowa, Canada

In this edition of Perspectives in Medical Education, Mirzazadeh and colleagues report on a longitudinal, evaluation study of curriculum change in their medical school [1]. They describe a four-phase process of reform framed by the Context, Input, Process and Product (CIPP) evaluation model [2] carried out at Tehran University of Medical Sciences, Iran.

We were particularly interested in the first of these: evaluating context in order to identify needs. Mirzazahed et al. report that they carried out a number of projects, combining qualitative and quantitative approaches to identify stakeholder perceptions of the quality and challenges in the existing programme, an assessment of the educational environment, and student performance measures [1]. We commend their efforts in terms of triangulating various data sources and it is clear that this approach gathered much data in terms of identifying areas of curricular content, delivery and assessment which would benefit from reform. However, as the focus of the paper is evaluative and atheoretical, we are concerned that their reform is likely to fall into the trap of being part of a socio-cultural reproduction exercise that results in repetition of sameness but no actual reform in the reform process [3].

To expand on this point, we suspect that any medical school, anywhere in the world, looking towards reform would identify similar themes if they asked the same questions and used the same data collection tools [3]. We were left feeling we knew nothing about curriculum reform at Tehran University of Medical Sciences. How does this differ from change at our own medical school, or that of the Chief Editor, or indeed any other institution? Surely the local context must have some relationship with the traditional curriculum and hence the nature of reform? If not, the same approaches to selection, teaching and/or assessment would look the same everywhere, which we know is not the case [4, 5]. This made us stop and think: what contextual features are currently neglected in the medical education literature but yet are influential, and hence important to consider in curriculum reform?

The list is probably endless but two concepts which are currently neglected in the medical education literature (yet not in other disciplines [6]) are the space and place of learning [7]. In health care (as opposed to health care education), space and place are generally discussed in relation to the importance of the built environment and its influence on patients and families’ experiences of their care [8]. There has been little research to date exploring this aspect of context on learner (and Faculty) educational experiences, yet emerging evidence indicates buildings are important in medical education generally, and curriculum reform specifically [9]. For example, consider the impact of having a fixed (non-adjustable) facility when planning change (perhaps one in dire need of modernization), versus the educational opportunities unleashed when designing new medical school buildings. What are the implications for reform of curricular delivery when the only facilities are fixed seating, tiered lecture theatres versus flexible room space which can be organized in different ways for different sessions [10]? Moreover, what is the social and cultural impact of having a medical school which is physically isolated from the rest of the university versus medical students sharing the same learning and social spaces as those from other disciplines?

In a number of research paradigms, the material environment (space) is frequently disregarded and reduced to an empty backdrop where it is simply presented as a fixed container of meaning, the stage or setting where the study takes place [11]. However, in socio-materiality literature the material environment, such as desks, chairs, technology, equipment and so on, is considered essential in order to understand the influence of the wider network of non-human factors on sociocultural phenomena such as professional identity formation. Space is not a static container in which to pour teachers and students, or a backdrop against which they perform [7]. The way in which spaces become specifically educational or learning spaces, how they are established in ways that enable or inhibit learning, how they create inequities or exclusions, open or limit possibilities for new practices and knowledge, and how space is represented in the artefacts we use in educational practices; in images and pictures are issues for medical education and demands in-depth consideration [12].

Similarly, place can shape sensibility and activity. Place can mean various things: from space (as above), to geographical location (e.g., country, region, town, area, building), to social setting (e.g., somewhere where everyday life activities happen such as a workplace or medical school), to a place which provides a strong sense of ‘belonging’ [13], and/or one which has a strong ethos or identity [14]. Place, therefore, is defined not only by geographic location and material form but also by the meaning and value that people associate with it and attach to it [15]. For example, our own (LH, JC) medical school is very proud of having a long and distinguished history of medicine, going back to 1495, and its location in a country where formal medical training dates back to the 1700s, whereas contemporary medical schools in the UK are equally proud of being new (and hence [implicitly] more forward thinking than the more venerable establishments!) and having a mission to increase diversity into medicine.

Bleakley et al. posit that ‘location, like power, can serve to shape or facilitate a dominant pattern of activity’ [16]. This refers to place being closely connected with both identity and power in terms of who controls access, who sets the agenda, whose interests are served, how those lower in the social hierarchy are treated [15, 16]. Place is thus fundamental to understanding knowledge production and dissemination, the ways in which place provides both the social settings/locations in which new ideas develop, and the attachment and sense of belonging one might have to a particular educational institution. Therefore, beyond simple location in space, from this perspective ‘place’ really matters for what we think theoretically as well as what we do practically [13].

As per the pessimistic French expression ‘*plus ça change, plus c’est la même*’ we translated for the title of this commentary, without a true appreciation of space and place, and hence their hidden influences [17], the effectiveness of educational change will be limited. Instead, we call for the use of theory to critically unpack the reproductive nature of curriculum reform, to make explicit the influence of local and national factors such as space and place on medical education and curriculum reform. Only by doing so will actual reform be possible.
